# Organellar Genome Analysis of the Red Alga *Acrosorium flabellatum* (Rhodophyta, Ceramiales, Delesseriaceae): Evidence of Gene Duplication and Group II Intron in *cox1* Gene

**DOI:** 10.3390/genes17101268

**Published:** 2026-10-12

**Authors:** Maheshkumar Prakash Patil, Jeong Gyu Kim, Young-Ryun Kim, Kyeongmin Kim, Pradip Vitthal Hirapure, Jong-Oh Kim, Kyunghoi Kim

**Affiliations:** 1 Department of Life Science, Parul Institute of Applied Sciences, Parul University, Vadodara 391760, India; maheshkumar.patil46404@paruluniversity.ac.in (M.P.P.); pradip.hirapure46370@paruluniversity.ac.in (P.V.H.); 2 School of Marine and Fisheries Life Science, Pukyong National University, Busan 48513, Republic of Korea; 3 Marine Eco-Technology Institute, Busan 48520, Republic of Korea; yykim@marine-eco.co.kr; 4 Faculty of Global Interdisciplinary Science and Innovation, Shizuoka University, 836 Oya, Suruga Ward, Shizuoka 422-8017, Japan; kim.kyeongmin@shizuoka.ac.jp; 5 Department of Microbiology, Pukyong National University, Busan 48513, Republic of Korea; 6 Department of Ocean Engineering, Pukyong National University, Busan 48513, Republic of Korea

**Keywords:** *Acrosorium flabellatum*, ceramiales, gene rearrangement, Group II intron, intronic *cox1*, mitochondrial genome, phylogenetic analysis, red algae

## Abstract

Mitochondrial genomes (mitogenome) offer important information about evolutionary history, genomic diversity, and structural dynamics of Rhodophytes. The complete mitochondrial genome of *Acrosorium flabellatum* Yamada, 1930 (Delesseriaceae, Ceramiales), is reported in this study. The circular mitogenome spans 31,818 bp and has a biased A+T (78.8%) composition. The mitogenome contains 61 genes, including two rRNAs, 33 tRNAs, 24 protein-coding genes (PCGs), and two open reading frames (ORFs). Notably, the mitogenome contains several duplicated genes (*nad6*, *sdh3*, *sdh2*) and a Group II intron within the *cox1* gene, suggesting continued intron mobility and structural expansion within Delesseriaceae. Codon usage indicated a strong preference for AT-rich codons, and intergenic regions were compact. A majority of PCGs showed positive GC skewness and negative AT skewness, reflecting conserved nucleotide bias across Ceramiales. Comparative analysis within the Ceramiales revealed lineage-specific differences in genome size, gene arrangement, gene content, with *A. flabellatum* revealing clear features of duplication and gene loss (*sdh4*, *rps3*). Several structural rearrangements, including gene order, presence of the Group II intron and ORFs, further support dynamic mitogenome evolution within Delesseriaceae. Phylogenetic analysis based on 23 conserved PCGs strongly supported the monophyly of Ceramiales and placed *A. flabellatum* within the Delesseriaceae clade with high bootstrap support. Overall, this study enriches the genomic resources of red algae, and sheds light on the comparative patterns of mitogenome structure within the Ceramiales. The mitogenome of *A. flabellatum* shows both conserved and dynamic features within this diverse red algal lineage.

## 1. Introduction

Red algae (phylum Rhodophyta) constitute one of the most ancient lineages of eukaryotic photosynthetic organisms, playing a pivotal role in marine ecosystems through their contributions to primary production, benthic habitat formation, and ecological diversity [1]. Characterized by a wide range of morphologies and complex life histories, red algae are taxonomically diverse, with the order Ceramiales standing out as one of the largest and most morphologically varied groups. This order includes several ecologically and economically significant families, such as Callithamniaceae, Ceramiaceae, Delesseriaceae, Rhodomelaceae, and Wrangeliaceae [2,3]. Investigating the mitochondrial genome architecture of the Ceramiales species is important for understanding their evolutionary histories, clarifying phylogenetic relationships, and lineage-specific molecular adaptations.

Red algal mitochondrial genomes are typically circular and compact, encoding a conserved set of genes involved in oxidative phosphorylation, protein synthesis, and RNA metabolism. Despite this conservation, considerable variation in genome size, gene content, and structural organization has been reported across red algal lineages, reflecting their distinct evolutionary histories [4,5]. These mitogenomes generally contain several rapidly evolving genes, which serve as informative markers for evolutionary and phylogenetic studies [4,6]. Within the subclass Rhodymeniophycidae, which includes the order Ceramiales, comparative analyses have revealed both rapid diversification and notable conservation of gene synteny among morphologically diverse multicellular taxa [4]. Such results highlight that red algal mitochondrial genomes are both flexible and conserved. In addition, using complete mitochondrial genomes and individual genes has greatly improved our ability to understand how red algae are related and how they have evolved over time [6,7].

A particularly intriguing feature of some red algal mitogenomes is the presence of Group II introns—self-splicing ribozymes that can excise themselves from precursor RNAs. These introns often harbor intron-encoded proteins, such as reverse transcriptase/maturases, which facilitate splicing and mobility [8,9]. In red algae, Group II introns have been identified in several mitochondrial genes, though their occurrence is sporadic and lineage-specific [10,11,12]. The insertion of a Group II intron within the *cox1* gene has been reported in multiple Florideophycean genera, such as *Ahnfeltia* [13], and *Grateloupia* [14,15,16,17], suggesting independent intron gain and loss events throughout evolutionary history. These mobile elements not only underscore the dynamic nature of organellar genomes but also serve as valuable molecular markers for evolutionary studies [10].

In addition to intron dynamics, gene rearrangements represent another significant aspect of mitogenome evolution within Ceramiales. Although many Florideophycean mitogenomes maintain a conserved gene order, several Ceramiales taxa exhibit substantial structural changes, including inversions, transpositions, and duplications [3,4,10,18]. These rearrangements are frequently linked to intron insertions, repetitive elements, and recombination hotspots, reflecting an ongoing process of mitochondrial genome reorganization and structural diversification across Ceramiales lineages [3,6,18,19,20,21,22].

In this study, we present the complete mitogenome of *A. flabellatum* Yamada 1930, a representative species of the family Delesseriaceae within the order Ceramiales. The objectives were to characterize the structural and functional features of the *A. flabellatum* mitogenome, and to determine its phylogenetic placement within Ceramiales using a concatenated dataset of conserved mitochondrial protein-coding genes. This work contributes to a deeper understanding of red algal mitogenome evolution, emphasizing the roles of intron mobility and gene-order variation in shaping mitochondrial diversity across Ceramiales.

## 2. Materials and Methods

### 2.1. Samples and DNA Extraction

In August 2024, we collected a sample of the red macroalga *A. flabellatum* from the coastal waters near Busan, South Korea (35°16′ N, 129°15′ E). This specimen was archived at the Ecological Restoration Group within the Marine Eco-Technology Institute in Busan, under the voucher ID PU-T01-S-MA-10 (contact: Dr. Young-Ryun Kim, yykim@marineeco.co.kr). Genomic DNA extraction (from thallus of specimen) was performed using the DNeasy Plant Kit (Qiagen, Hilden, Germany), adhering to the manufacturer’s recommended protocol. The DNA concentration was measured with a NanoDrop spectrophotometer (Thermo Fisher Scientific D1000, Waltham, MA, USA), and the purified DNA was stored at −20 °C for future applications.

### 2.2. Library Preparation, Sequencing, and Mitogenome Assembly

Genomic DNA was processed using the TruSeq Nano DNA Library Preparation Kit (Illumina, San Diego, CA, USA) and sequenced at Macrogen Inc. (Daejeon, Republic of Korea; https://www.macrogen.com/ko/) on the Illumina HiSeq 2500 platform. Sequencing was performed with paired-end reads (2 × 150 bp), targeting an average insert size of 350 bp. To enhance data quality, reads with a Phred score below 20 and adapter contaminants were filtered out using Trimmomatic v0.38 [23]. A subset of the high-quality reads was randomly selected for *de novo* mitochondrial genome assembly. Read quality was assessed using FastQC v0.11.7 [24]. Assembly of the *A. flabellatum* mitogenome was conducted with NOVOPlasty v4.3.5 [25], utilizing Illumina short-read data. The mitogenome of *Dasya binghamiae* (GenBank accession: KX247283), a member of the Dasyaceae family, served as the reference sequence.

### 2.3. Mitogenome Annotation and Analysis

The assembled mitogenome was compared against existing mitogenome sequences in the NCBI database using BLAST. Annotation was performed using MFannot [26], applying Translation Table 4. Protein-coding genes (PCGs) were predicted with an open reading frame (ORF) Finder and validated through BLAST searches against the NCBI protein database [27]. Transfer RNA (tRNA) genes were identified using tRNAscan-SE 2.0 [28], and RNAweasel was employed to confirm RNA gene locations and detect intronic regions [29,30]. A circular representation of the mitochondrial genome was generated using Figures and Tables v1.3.1 [31]. Nucleotide composition and relative synonymous codon usage (RSCU) (excluding stop codon) were calculated using MEGA11 v11.2.8 [32]. Strand asymmetry was evaluated using the formulas GC-skew = (G − C)/(G + C) and AT-skew = (A − T)/(A + T) [33]. Comprehensive analyses—including annotation, BLAST, RNA identification, tandem repeat detection, RSCU analysis, and phylogenetic reconstruction—were conducted using web-based tools during February to July 2025.

### 2.4. Phylogenetic Analysis

To determine the phylogenetic position of *A. flabellatum* within the phylum Rhodophyta, a comprehensive phylogenetic analysis was performed using complete mitogenome sequences. A total of 35 mitogenomes, including that of *A. flabellatum*, were retrieved from GenBank database, following the updated phylogenetic framework of the order Ceramiales [3]. Among these, 34 species were designated as the ingroup, while *Rhodymenia pseudopalmata* was selected as the outgroup (Table 1). The analysis focused on 23 conserved mitochondrial PCGs, comprising seven NADH dehydrogenase subunits (*nad1–6*, *nad4L*), four ATP synthase subunits (*atp4*, *atp6*, *atp8*, *atp9*), four ribosomal proteins *(rps3*, *rps11*, *rps12*, *rpl16*), three cytochrome oxidase subunits (*cox1–3*), three succinate dehydrogenase subunits (*sdh2–4*), apocytochrome b (*cob*), and a secY-independent protein translocase (*tatC*). The nucleotide sequences of these genes were concatenated and aligned using MAFFT v7.0 [34]. The best-fitting substitution model was determined using ModelFinder implemented in IQ-TREE, based on the Bayesian Information Criterion (BIC), which identified GTR+F+I+G4 as the optimal model [35]. Phylogenetic inference was carried out using the maximum likelihood (ML) method in IQ-TREE [36], and branch support values were assessed through 1000 ultrafast bootstrap replicates to evaluate node reliability. The resulting ML tree was visualized using the Interactive Tree of Life (iTOL) v7 web interface [37].

## 3. Results

### 3.1. Genome Structure, Organization and Composition

The genomic library of *A. flabellatum* was sequenced using the Illumina platform, yielding a total of 3,444,461,604 raw bases, and 22,811,004 total reads. These reads exhibited a GC content of 45.4%, with high accuracy in Q20 and Q30 scores of 98.3% and 93.2%, respectively. Following quality filtering to remove low-confidence sequences, 3,189,090,087 filtered bases, and 21,149,740 total reads were retained, showing improved metrics with a GC content of 45.1%, Q20 accuracy of 99.4%, and Q30 accuracy of 95.8%. The filtered reads were then assembled *de novo*, achieving complete coverage of the mitochondrial genome with an average sequencing depth of 252.5×. The insert size was estimated at 416.5 bp with a standard deviation of 98.2 bp.

**Figure 1:**
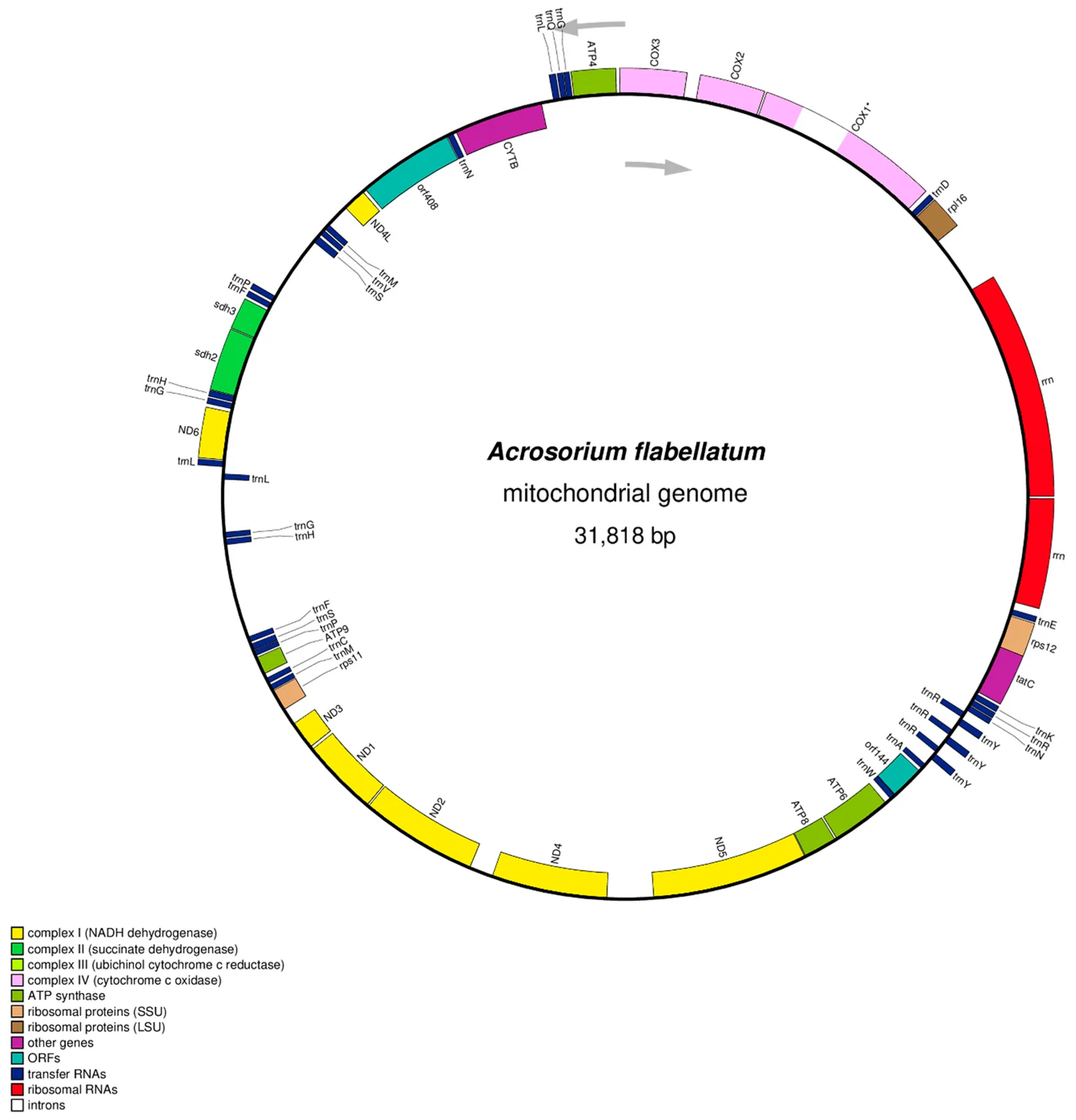
Circular mitochondrial genome of *A. flabellatum* (GenBank accession number PX111276). Arrows indicate gene orientation, and colors represent functional gene groups with their corresponding acronyms.

The complete mitogenome of *A. flabellatum* was successfully assembled as a circular DNA molecule measuring 31,818 bp in length (Figure 1) and has been deposited in GenBank under the accession number PX111276. Genome annotation identified 61 genes in total, including 24 PCGs, 33 tRNAs, two rRNAs, and two ORFs. Gene distribution analysis revealed that the heavy (H) strand encoded 10 PCGs, 12 tRNAs, and both rRNAs, whereas the light (L) strand contained 14 PCGs, 21 tRNAs, and the two ORFs. The nucleotide composition of the mitogenome showed a pronounced A+T bias (78.8%), with individual base proportions of 41.1% adenine (A), 37.7% thymine (T), 10.8% guanine (G), and 10.4% cytosine (C) (Table 2). Both AT skew (0.043) and GC skew (0.023) were positive, indicating a compositional tendency toward A over T and G over C across the mitogenome.

### 3.2. Intergenic Regions and Codon Usage

Analysis of the intergenic regions in the mitochondrial genome of A. flabellatum revealed a single case of gene overlap, consisting of a 125 bp overlap between the tatC and rps12 genes. Apart from this overlap, intergenic spacer regions varied in length from 4 to 835 bp, with overlaps ranging from 0 to 125 bp. The total cumulative size of the intergenic regions was 2239 bp, accounting for 7.04% of the complete mitogenome. The largest intergenic region, measuring 835 bp, was located between the *trnS* and *trnP* genes (Table 2).

**Figure 2:**
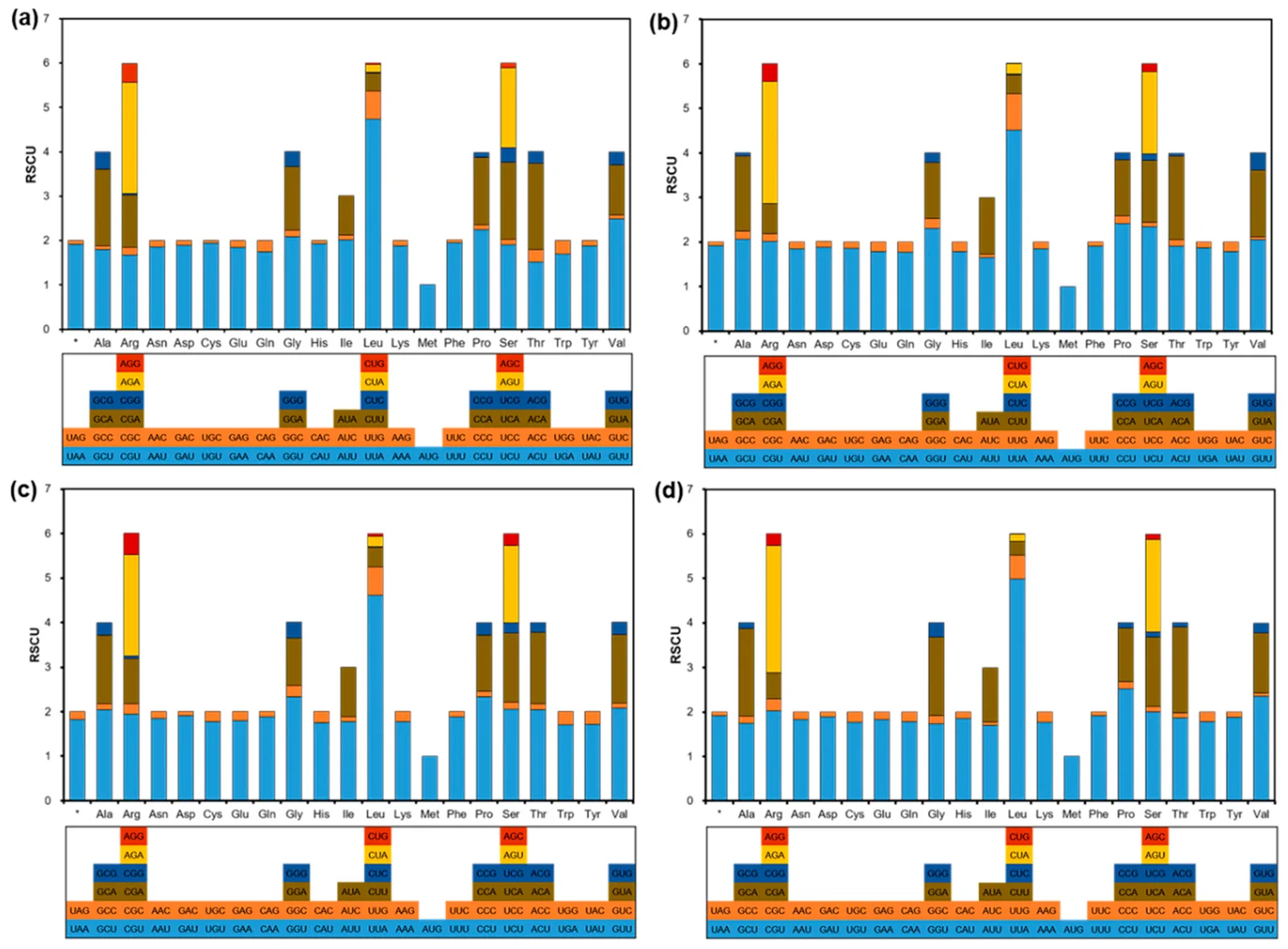
Relative synonymous codon usage (RSCU) patterns of mitochondrial PCGs across four Delesseriaceae species: (**a**) *A. flabellatum*, (**b**) *D. binghamiae*, (**c**) *D. japonica*, and (**d**) *H. pulchra.* * indicates stop codon.

The RSCU patterns of mitochondrial PCGs in *A. flabellatum* were analyzed and compared with those of three other species within the Delesseriaceae family (Figure 2). The PCGs of *A. flabellatum* encoded a total of 6663 amino acids, with codons for leucine being the most abundant (1016 residues; 15.25%), followed by isoleucine (859; 12.89%), phenylalanine (800; 12.01%), serine (476; 7.14%), and asparagine (434; 6.51%). These results reveal a distinct codon usage bias favoring specific amino acids. Notably, the codon UUA (leucine) was the most frequently used, appearing 802 times and exhibiting the highest RSCU value of 4.74, indicating a strong preference. In contrast, AUG (methionine) was the only codon with an RSCU value of one, reflecting no bias. Overall, 29 codons showed RSCU values greater than one, suggesting preferential usage, while 34 codons had values below one, excluding stop codon, indicating less-frequent use.

### 3.3. Protein-Coding Genes

**Figure 3:**
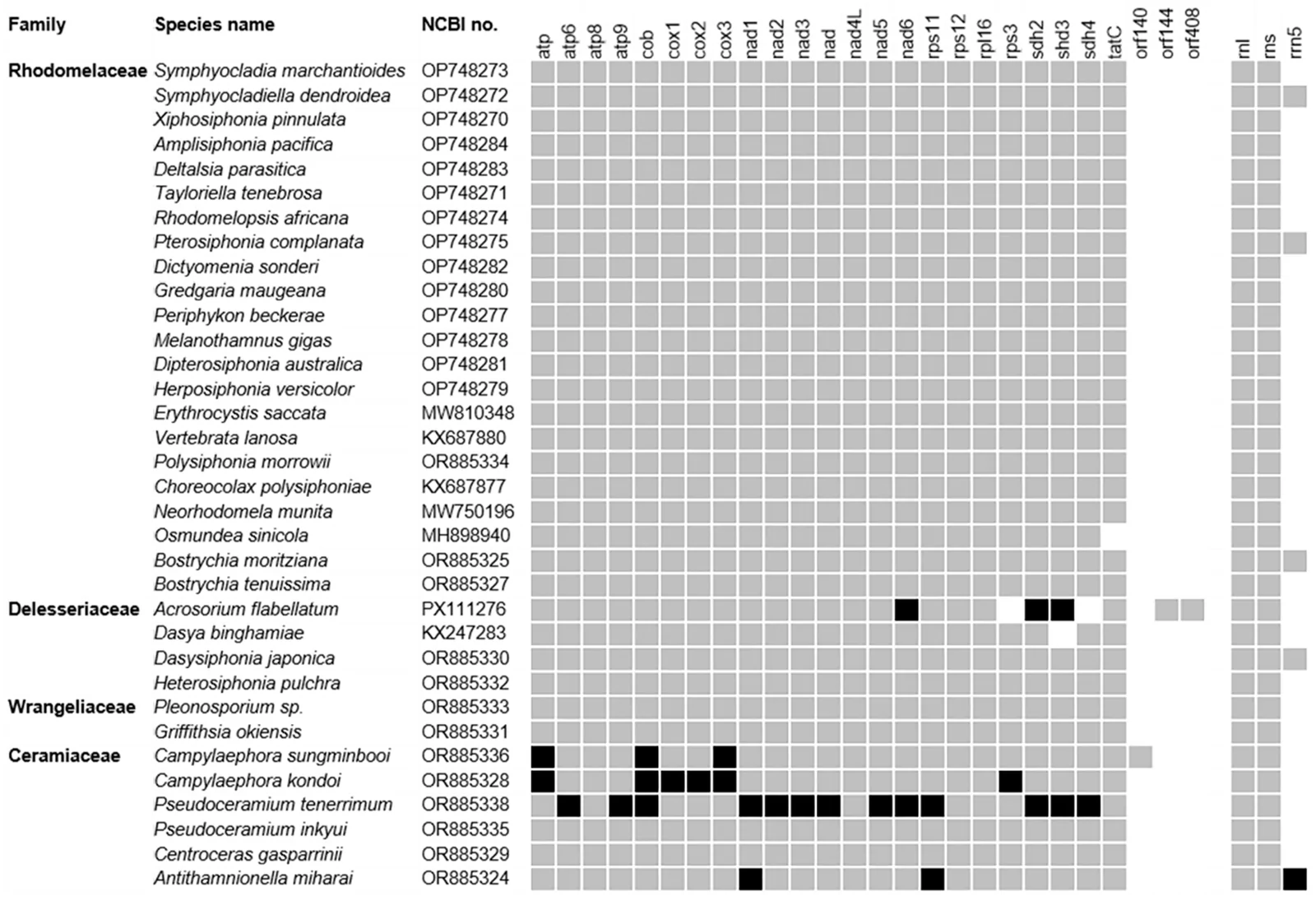
Presence and absence of mitochondrial protein-coding genes and ribosomal RNAs in Ceramiales species. Gray indicates presence, black indicates duplicated copies, and white indicates absence.

A core set of 23 PCGs was consistently conserved across all examined families within Ceramiales. In contrast, species belonging to Ceramiaceae displayed the greatest genomic complexity, containing up to 36 PCGs (Table 1, Figure 3). The PCGs of the *A. flabellatum* mitogenome spans 19,989 bp, representing 62.82% of the total mitogenome sequence. These coding regions exhibit a strong A+T bias and negative AT-skew values, as summarized in Table 3. A total of 24 PCGs plus two predicted ORFs were annotated. Among these genes, *nad5* was the longest (1989 bp), whereas *atp9* showed the shortest length (231 bp) (Table 4). Nucleotide composition analysis across individual PCGs demonstrated consistent A+T dominance. Although most genes exhibited negative AT skewness, except *rpl16*, *orf408*, and *rps11* showed positive AT-skew values. In contrast, GC-skew values were positive, with the exception of *orf408*, which had a negative GC-skew value (Table 4). Evaluation of codon usage further revealed a strong preference for ATG as the start codon and TAA as the stop codon. Only a few deviations were observed: *rps11* and *tatC* initiated with GTG, while *atp8* terminated with TAG (Table 1). Several notable structural and functional genomic features were identified in the *A. flabellatum* mitogenome. These include a 629 bp insertion within the *cox1* gene that may represent a putative Group II intron, duplication of genes such as *nad6*, *sdh2*, and *sdh3*, and the detection of two putative ORFs (*orf408* and *orf144*). In contrast, some genes typical of other Ceramiales mitogenomes such as *sdh4* and *rps3* were not detected in this assembly (Figure 3).

### 3.4. Transfer and Ribosomal RNA Genes

Most Ceramiales mitogenomes contained two ribosomal RNA genes. However, copy number variation was observed in several taxa, where the total number of rRNA genes increased to three or four, primarily due to the presence of additional 5S rRNA gene copies (Table 1, Figure 3). The mitogenome of *A. flabellatum* encodes the typical pair of rRNA genes: the small subunit *rns* (1323 bp) and the large subunit *rnl* (2729 bp), both positioned on the H-strand. Collectively, these rRNAs span 4052 bp, representing 12.73% of the total genome length (Table 2). In addition, a complete set of 33 tRNA genes was identified, totaling 2544 bp and accounting for approximately 8% of the mitogenome. The tRNAs ranged in size from 72 bp (*trnG*, *trnE*) to 94 bp (*trnS*), and none contained intronic insertions. Analysis of anticodon usage revealed standard correspondence between codons and anticodons, consistent with patterns observed in other Ceramiales species. Notably, five tRNA genes (*trnG*, *trnL*, *trnN*, *trnR*, and *trnS*) were each represented by two distinct anticodons, suggesting redundancy and enhanced flexibility in mitochondrial codon recognition in *A. flabellatum* (Table 2).

### 3.5. Gene Rearrangement

Comparative mitogenomic analysis across Ceramiales revealed clear differences in both genome size and structural organization among the examined families (Table 1; Figure 3). Ceramiaceae species showed the largest mitogenomes, which corresponded with the presence of duplicated genes, additional open reading frames (ORFs) and rRNA genes, and intron insertions. The newly sequenced mitogenome of *A. flabellatum*, a member of Delesseriaceae, also revealed several signatures of genomic expansion and rearrangements. The circular mitogenome measures 31,818 bp and contains two ORFs (*orf408*, *orf144*), along with duplicated copies of *nad6*, *sdh2*, and *sdh3*, contributing to increased gene content (Table 2; Figure 1). A Group II intron within the *cox1* gene was additionally identified, highlighting the presence of mobile intronic elements that may facilitate structural rearrangement, whereas *sdh4* and *rps3* genes typically conserved in other Ceramiales mitogenomes were not identified in *A. flabellatum*, indicating potential lineage-specific gene loss. Together, these features support a dynamic evolutionary trajectory for the Delesseriaceae mitochondrial genome and emphasize the structural distinctiveness of *A. flabellatum* within Ceramiales.

### 3.6. Phylogeny

**Figure 4:**
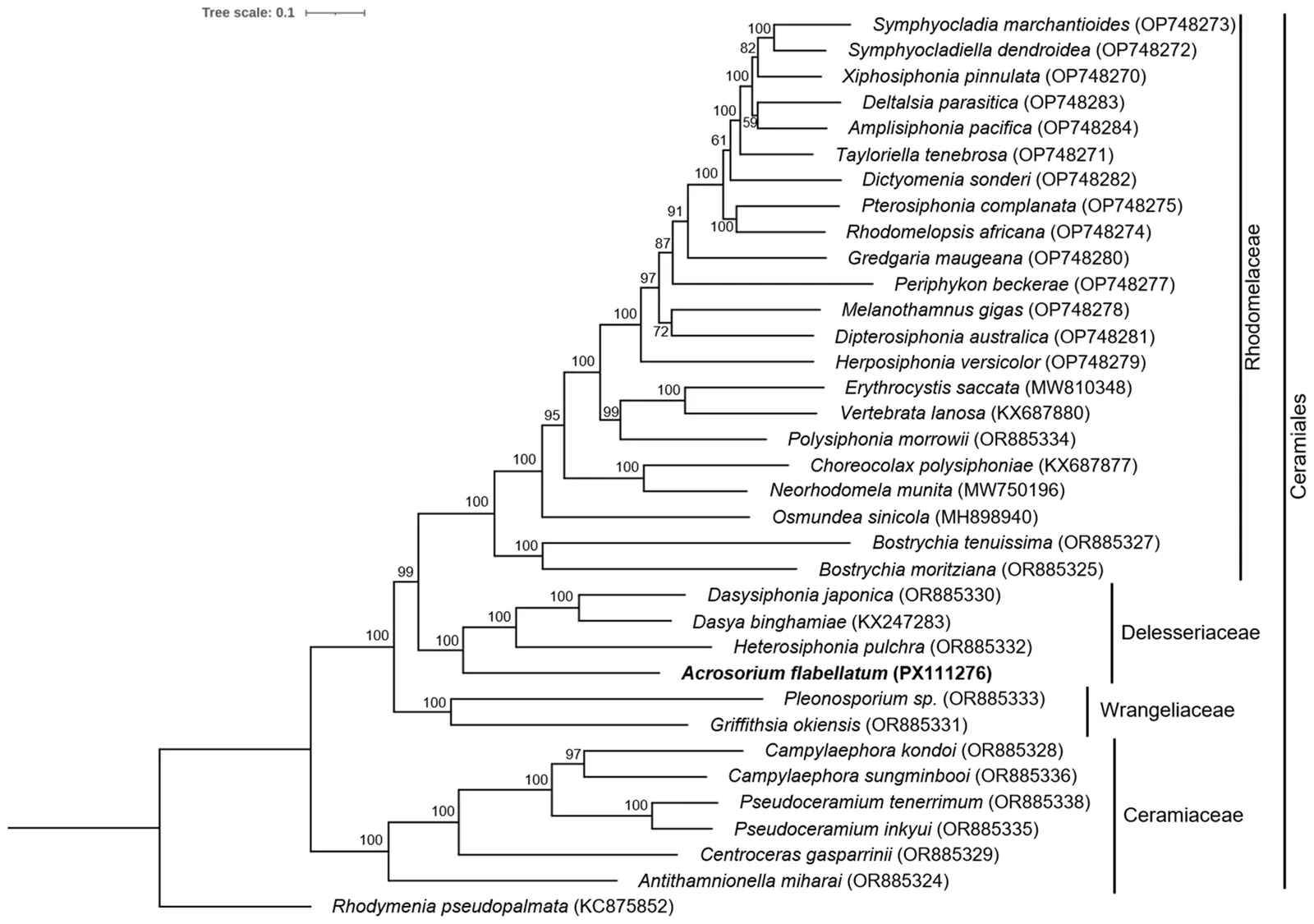
Maximum likelihood phylogenetic tree of the order Ceramiales constructed from a concatenated alignment of 23 conserved PCGs. The tree was rooted with *R. pseudopalmata* (Rhodymeniaceae) as the outgroup. Bootstrap support values (%) are shown at each node. Newly generated sequence from this study is indicated in bold. GenBank accession numbers are provided in parentheses following the species names.

Phylogenomic analysis was conducted using a concatenated alignment of 23 conserved PCGs, excluding ORFs and duplicated genes (Figure 4). The resulting ML tree clearly indicates the familial relationships within the order Ceramiales. The order was identified as monophyletic and comprised four different families, namely Ceramiaceae, Delesseriaceae, Rhodomelaceae, and Wrangeliaceae. Delesseriaceae and Rhodomelaceae formed a well-supported sister clade and were revealed to be closely related to Wrangeliaceae, while Ceramiaceae represented the earliest diverging lineage. The tree was rooted with Rhodymenia pseudopalmata, an outgroup species, providing a reference for divergence. In this study, sequenced *A. flabellatum* clustered within the Delesseriaceae clade with strong bootstrap support. Its position supports its taxonomic identity and close evolutionary relationship with other Delesseriaceae taxa. These results support the phylogenetic distinctiveness of *A. flabellatum* within the red algal lineage.

## 4. Discussion

The mitogenome of *A. flabellatum* revealed many features consistent with other Ceramiales mitogenomes, including a circular architecture, biased AT composition, and positive AT and GC skewness. However, the genome length (31,818 bp) is larger than many typical Delesseriaceae mitogenomes (Table 1), reflecting an apparent expansion in this lineage [3,18]. This increase is due to the additional ORFs, duplicated genes, and intronic insertions, all of which contribute to increased genome complexity in *A. flabellatum*. Positive AT and GC skewness values in *A. flabellatum* indicate strand-specific nucleotide preferences, a pattern also observed in other Delesseriaceae species (Table 1) [3].

Intergenic regions of *A. flabellatum* comprise a minor fraction of the total mitogenome, featuring a single gene overlap and the longest gap between two tRNA genes. This compact organization feature is consistent with the other Rhodophytes, where intergenic regions are mostly associated with gene duplications and insertion of introns [6,10,29]. RSCU analysis revealed a preference for the start codon ATG and the stop codon TAA (Figure 2, Table 5). Similar trends reported in other red algal mitogenomes suggest that translational selection influences codon usage patterns in Ceramiales [5,17].

The PCG composition of *A. flabellatum* shows both conserved features and lineage-specific evolution within Ceramiales. Overall, its gene content is broadly consistent with members of Rhodomelaceae, Wrangeliaceae, and Delesseriaceae; however, *A. flabellatum* revealed several distinct features, like additional ORFs (*orf408* and *orf144*), duplicated copies of genes (*nad6*, *sdh2*, *sdh3*), and the absence of *sdh4* and *rps3* genes (Figure 3, Table 5). The presence of new ORFs and several gene duplications is a common pattern observed in Ceramiaceae mitogenomes [3,4]. These duplications contribute to genome expansion and support the continuous mitogenome reorganization is ongoing in this lineage [3,4]. Likewise, the gene losses observed in *A. flabellatum* reflect the trends previously reported in other Ceramiales taxa, such as the loss of the *tatC* gene in *O. sinicola* [22] and the *sdh3* gene in *D. binghamiae* [18]. Such lineage-specific losses and gains are recurring characteristics of red algal mitochondrial evolution within Delesseriaceae [3,6,18]. A Group II intron was identified within the *cox1* gene of *A. flabellatum*, representing a distinctive feature of its mitogenome and constituting the first report of such an insertion within the Ceramiales (Table 1). Group II introns are mobile, self-splicing elements known to contribute to mitochondrial genome expansion and structural variability [9,10,12]. Such type of introns has been reported sporadically across red algal lineages, but no intronic *cox1* gene has been reported in any Delesseriaceae species, making this finding particularly significant. However, *cox1* introns are mostly reported within Florideophyceae, their distribution is remarkably inconsistent, with frequent independent gain and loss events across closely related taxa [4,6,11,12,13,14,15,16,17]. Comparable intronic insertions have been associated with lineage-specific genome restructuring in other red algae, highlighting the evolutionary relevance of these mobile elements as drivers of genomic diversity in Ceramiales [6,12,29].

The rRNA and tRNA gene complement of *A. flabellatum* is largely conserved when compared with other Ceramiales species (Table 1). Its mitogenome contains the typical two rRNA genes (*rns*, *rnl*) located on the H-strand, although some Ceramiales taxa possess additional or duplicated rRNA copies—specifically 1–2 copies of *rns5* [3,21]. In contrast, *A. flabellatum* contains a complete set of 33 tRNA genes, with higher tRNA counts compared to other Ceramiales species, which generally range from 18 to 28 [3,6,18,19,20,21,22]. This expanded tRNA count also contributes substantially to the larger mitogenome size observed in *A. flabellatum*. While anticodon usage is broadly conserved, the presence of multiple anticodon variants for several tRNAs (*trnG*, *trnL*, *trnN*, *trnR*, *trnS*) suggests functional redundancy and greater flexibility in codon recognition. Similar patterns are observed in other Delesseriaceae mitogenomes, where increased anticodon diversity may enhance translational efficiency and adaptability. Such variation in tRNA copy number and anticodon sets within Ceramiales likely indicates lineage-specific fine-tuning of mitochondrial translation processes [4,6].

Genome rearrangements are a defining feature of Delesseriaceae mitogenomes, and *A. flabellatum* shows clear evidence of evolution. The presence of a Group II intron in *cox1*, duplicated genes (*nad6*, *sdh2*, *sdh3*) and additional ORFs (*orf408*, *orf144*) mark this species as the first Delesseriaceae member with this specific combination of rearrangement features. These modifications distinguish Delesseriaceae from Rhodomelaceae and Wrangeliaceae families, which typically retain more conserved gene orders [3,6,18,19,20,21,22].

Phylogenomic studies provide important insights into the evolutionary relationships within Ceramiales families, and the topology recovered in this study aligns well with earlier mitochondrial and plastid-based reconstructions [3]. Ceramiales formed a strongly supported monophyletic group, resolving into four major lineages: Ceramiaceae, Wrangeliaceae, Delesseriaceae, and Rhodomelaceae. *A. flabellatum* clustered within the Delesseriaceae, with high bootstrap support confirming its taxonomic position and evolutionary affinity with other species of the family. The recovered sister relationship between Delesseriaceae and Rhodomelaceae, along with the intermediate placement of Wrangeliaceae, is a pattern similar to those reported in earlier phylogenomic studies [6,18]. In contrast, Ceramiaceae appeared as the earliest diverging lineage, indicating an ancient split from the other families—consistent with previous mitochondrial and multigene studies [3,4]. In this study, the expanded gene set, intron insertion, and genome rearrangements in *A. flabellatum* provide useful phylogenetic signals that support its distinct position within Delesseriaceae. These features highlight the dynamic nature of mitochondrial evolution in Ceramiales and underscore the value of *A. flabellatum* as a newly characterized representative of the family.

The mitochondrial phylogeny recovered in this study provides useful insights into genome evolution within Ceramiales and aligns well with earlier mitochondrial and plastid-based reconstructions [3]. Ceramiales formed a strongly supported monophyletic group, resolving into four major lineages: Ceramiaceae, Wrangeliaceae, Delesseriaceae, and Rhodomelaceae. *A. flabellatum* was recovered within the sampled Delesseriaceae lineage, with high bootstrap support reflecting its mitochondrial phylogenetic affinity with other members of the family. The sister relationship between Delesseriaceae and Rhodomelaceae, along with the intermediate placement of Wrangeliaceae, is consistent with patterns reported in earlier mitochondrial phylogenomic studies [6,18]. In contrast, Ceramiaceae appeared as the earliest diverging lineage, indicating an ancient split from the other families, consistent with previous mitochondrial and multigene analyses [3,4]. In this study, the expanded gene set, intron insertion, and genome rearrangements in *A. flabellatum* provide phylogenetic signals supporting its distinct position within Delesseriaceae. These features highlight the dynamic nature of mitochondrial genome evolution in Ceramiales and underscore the value of *A. flabellatum* as a newly characterized representative of the family.

## 5. Conclusions

This study presents the first report on the complete mitogenome of *A. flabellatum* (Delesseriaceae, Ceramiales), which showed both conserved and dynamic evolutionary features. The 31,818 bp circular mitogenome has the typical red algal gene complement but also includes unique features such as duplicated genes (*sdh2*, *sdh3*, and *nad6*), two ORFs (*orf408* and *orf144*), and a *cox1* gene with Group II intron. These characteristics indicate active genome expansion and rearrangement within Delesseriaceae. Comparative mitogenomic analysis revealed a conserved core structure within Ceramiales, while lineage-specific gene losses and duplications enhance mitochondrial diversity. The presence of novel ORFs and several gene duplications is a common pattern in Ceramiaceae mitogenomes, yet this study represents the first report of such features within the Delesseriaceae. Phylogenomic analysis confirms the monophyly of Ceramiales and classified *A. flabellatum* within Delesseriaceae. The mitogenome of *A. flabellatum* enhances comparative genomic resources for red algae and provides a foundation for future investigations into mitochondrial evolution, intron dynamics, and phylogenomics across red algae.

## Figures and Tables

**Table 1:** The Ceramiales species complete mitogenome sequences used in this study.

Family	Species	NCBI No.	Size (bp)	Total Genes	PCG	tRNA	rRNA	ORF	Gene with Intron	A+T (%)	G+C (%)	AT Skew	GC Skew	Ref.
Rhodomelaceae	*Symphyocladia marchantioides*	OP748273	25,661	47	23	22	2	0	*trnI1*, *trnI2*	75.5	24.5	0.062	0.031	[21]
	*Symphyocladiella dendroidea*	OP748272	24,959	44	23	18	3	0	*trnI*, *trnS*	76.8	23.2	0.062	0.033	[21]
	*Xiphosiphonia pinnulata*	OP748270	25,531	48	23	23	2	0	*trnI*, *trnS*	77.2	22.8	0.056	0.037	[21]
	*Amplisiphonia pacifica*	OP748284	25,267	48	23	23	2	0	*trnI*, *trnS*	76.3	23.7	0.066	0.025	[21]
	*Deltalsia parasitica*	OP748283	25,071	45	23	20	2	0	*trnI*, *trnS*	76.6	23.4	0.065	0.030	[21]
	*Tayloriella tenebrosa*	OP748271	25,533	47	23	22	2	0	*trnI*	76.3	23.7	0.070	0.025	[21]
	*Rhodomelopsis africana*	OP748274	26,394	47	23	22	2	0	*trnI*	75.5	24.5	0.052	0.022	[21]
	*Pterosiphonia complanata*	OP748275	26,306	46	23	20	3	0	*trnI*	75.4	24.6	0.053	0.041	[21]
	*Dictyomenia sonderi*	OP748282	25,804	51	23	26	2	0	*trnI*, *trnS*	75.9	24.1	0.063	0.032	[21]
	*Gredgaria maugeana*	OP748280	25,378	46	23	21	2	0	*trnI*	76.7	23.2	0.062	0.041	[21]
	*Periphykon beckerae*	OP748277	25,139	48	23	23	2	0	*trnI*	76.5	23.5	0.057	0.015	[21]
	*Melanothamnus gigas*	OP748278	25,445	49	23	24	2	0	*trnI*, *trnS*	75.6	24.4	0.062	0.022	[21]
	*Dipterosiphonia australica*	OP748281	25,142	48	23	23	2	0	*trnE*, *trnR*	76.2	23.8	0.069	0.031	[21]
	*Herposiphonia versicolor*	OP748279	24,716	48	23	23	2	0	*trnI*	76.8	23.2	0.059	0.024	[21]
	*Erythrocystis saccata*	MW810348	24,975	45	23	20	2	0	*-*	76.6	23.4	0.055	0.017	[19]
	*Vertebrata lanosa*	KX687880	25,337	44	23	19	2	0	*trnS*	76.4	23.6	0.060	0.011	[6]
	*Polysiphonia morrowii*	OR885334	25,235	46	23	21	2	0	*-*	76.1	23.9	0.059	0.021	[3]
	*Choreocolax polysiphoniae*	KX687877	25,357	45	23	20	2	0	*-*	79.4	20.6	0.057	0.030	[6]
	*Neorhodomela munita*	MW750196	25,318	48	23	23	2	0	*-*	74.9	25.1	0.059	0.027	[20]
	*Osmundea sinicola*	MH898940	25,021	44	22	20	2	0	*-*	76.8	23.2	0.050	0.025	[22]
	*Bostrychia moritziana*	OR885325	25,508	44	23	18	3	0	*-*	74.4	25.6	0.053	0.006	[3]
	*Bostrychia tenuissima*	OR885327	25,423	44	23	19	2	0	*-*	72.1	27.9	0.053	0.009	[3]
Delesseriaceae	*A*. *flabellatum*	PX111276	31,818	61	24	33	2	2	*cox1*	78.8	21.2	0.043	0.023	This study
	*D. binghamiae*	KX247283	26,052	46	22	22	2	0	*-*	77.4	22.6	0.043	0.009	[18]
	*Dasysiphonia japonica*	OR885330	25,918	48	23	22	3	0	*-*	77.0	23.0	0.045	0.014	[3]
	*Heterosiphonia pulchra*	OR885332	25,591	46	23	21	2	0	*-*	77.9	22.0	0.045	0.011	[3]
Wrangeliaceae	*Pleonosporium* sp.	OR885333	24,963	47	23	22	2	0	*-*	74.1	25.8	0.046	0.013	[3]
	*Griffithsia okiensis*	OR885331	24,865	45	23	20	2	0	*-*	77.9	22.1	0.058	0.042	[3]
Ceramiaceae	*Campylaephora sungminbooi*	OR885336	27,664	54	27	24	2	1	*-*	70.7	29.3	0.071	0.025	[3]
	*Campylaephora kondoi*	OR885328	31,430	56	29	25	2	0	*-*	69.8	30.2	0.078	−0.002	[3]
	*Pseudoceramium tenerrimum*	OR885338	36,678	65	36	27	2	0	*-*	71.5	28.5	−0.008	0.022	[3]
	*Pseudoceramium inkyui*	OR885335	25,516	47	23	22	2	0	*-*	71.4	28.6	0.064	0.030	[3]
	*Centroceras gasparrinii*	OR885329	24,915	47	23	22	2	0	*-*	72.4	27.6	0.052	0.033	[3]
	*Antithamnionella miharai*	OR885324	27,190	57	25	28	4	0	*-*	71.7	28.3	−0.068	0.033	[3]

**Table 2:** Features of the mitogenome of *A. flabellatum*.

Gene Name	Start	End	Length (bp)	Amino Acids	Start Codon	Stop Codon	Anticodon	Strand	Gap/Overlap
*rnl*	1	2729	2729	-	-	-	-	H	737
*rpl16*	3467	3877	411	136	ATG	TAA	-	H	10
*trnD*	3888	3961	74	-	-	-	GTC	H	53
*cox1*	4015	6254	2240	536	ATG	TAA	-	H	18
*exon-1*	4015	5167	1153	-	-	-	-	H	-
*Intron*	5168	5796	629	-	-	-	-	H	-
*exon-2*	5797	6254	458	-	-	-	-	H	-
*cox2*	6273	7064	792	263	ATG	TAA	-	H	151
*cox3*	7216	8025	810	269	ATG	TAA	-	H	37
*atp4*	8063	8602	540	179	ATG	TAA	-	H	15
*trnG*	8618	8689	72	-	-	-	TCC	H	4
*trnQ*	8694	8766	73	-	-	-	TTG	H	13
*trnL*	8780	8863	84	-	-	-	TAA	L	152
*cob*	9016	10,155	1140	379	ATG	TAA	-	L	40
*trnN*	10,196	10,276	81	-	-	-	ATT	L	7
*orf408*	10,284	11,510	1227	408	ATG	TAA	-	L	39
*nad4L*	11,550	11,852	303	100	ATG	TAA	-	L	295
*trnM*	12,148	12,223	76	-	-	-	CAT	L	16
*trnV*	12,240	12,312	73	-	-	-	TAC	L	31
*trnS*	12,344	12,437	94	-	-	-	GCT	L	835
*trnP*	13,273	13,345	73	-	-	-	TGG	H	27
*trnF*	13,373	13,445	73	-	-	-	GAA	H	31
*sdh3*	13,477	13,863	387	128	ATG	TAA	-	H	8
*sdh2*	13,872	14,633	762	253	ATG	TAA	-	H	4
*trnH*	14,638	14,713	76	-	-	-	GTG	H	14
*trnG*	14,728	14,801	74	-	-	-	GCC	H	39
*nad6*	14,841	15,452	612	203	ATG	TAA	-	H	10
*trnL*	15,463	15,537	75	-	-	-	TAG	H	84
*trnL*	15,622	15,696	75	-	-	-	TAG	L	10
*nad6*	15,707	16,318	612	203	ATG	TAA	-	L	39
*trnG*	16,358	16,431	74	-	-	-	GCC	L	14
*trnH*	16,446	16,521	76	-	-	-	GTG	L	4
*sdh2*	16,526	17,287	762	253	ATG	TAA	-	L	8
*sdh3*	17,296	17,682	387	128	ATG	TAA	-	L	31
*trnF*	17,714	17,786	73	-	-	-	GAA	L	25
*trnS*	17,812	17,896	85	-	-	-	TGA	L	4
*trnP*	17,901	17,973	73	-	-	-	TGG	L	21
*atp9*	17,995	18,225	231	76	ATG	TAA	-	L	57
*trnC*	18,283	18,355	73	-	-	-	GCA	L	25
*trnM*	18,381	18,453	73	-	-	-	CAT	L	9
*rps11*	18,463	18,741	279	92	GTG	TAA	-	L	211
*nad3*	18,953	19,318	366	121	ATG	TAA	-	L	34
*nad1*	19,353	20,339	987	328	ATG	TAA	-	L	18
*nad2*	20,358	21,857	1500	499	ATG	TAA	-	L	298
*nad4*	22,156	23,634	1479	492	ATG	TAA	-	L	585
*nad5*	24,220	26,208	1989	662	ATG	TAA	-	L	6
*atp8*	26,215	26,643	429	142	ATG	TAG	-	L	21
*atp6*	26,665	27,432	768	255	ATG	TAA	-	L	74
*trnW*	27,507	27,581	75	-	-	-	TCA	L	0
*orf144*	27,582	28,016	435	144	ATG	TAA	-	L	38
*trnA*	28,055	28,127	73	-	-	-	TGC	L	94
*trnY*	28,222	28,311	90	-	-	-	GTA	L	16
*trnR*	28,328	28,404	77	-	-	-	TCT	L	90
*trnY*	28,495	28,584	90	-	-	-	GTA	L	16
*trnR*	28,601	28,677	77	-	-	-	TCT	L	90
*trnY*	28,768	28,857	90	-	-	-	GTA	L	16
*trnR*	28,874	28,950	77	-	-	-	TCT	L	31
*trnN*	28,982	29,055	74	-	-	-	GTT	H	7
*trnR*	29,063	29,136	74	-	-	-	ACG	H	12
*trnK*	29,149	29,223	75	-	-	-	TTT	H	27
*tatC*	29,251	30,003	753	250	GTG	TAA	-	H	−125
*rps12*	29,879	30,295	417	138	ATG	TAA	-	H	12
*trnE*	30,308	30,379	72	-	-	-	TTC	H	93
*rns*	30,473	31,795	1323	-	-	-	-	H	23

**Table 3:** Nucleotide composition of mitogenomes in four Delesseriaceae species.

Name	Length (bp)	Nucleotide Composition (%)						AT Skew	GC Skew
		A	T	G	C	A+T	G+C		
Whole genome									
*A*. *flabellatum*	31,818	41.1	37.7	10.8	10.4	78.8	21.2	0.043	0.023
*D. binghamiae*	26,052	40.3	37.0	11.4	11.2	77.4	22.6	0.043	0.009
*D. japonica*	25,918	40.2	36.8	11.7	11.3	77.0	23.0	0.045	0.014
*H*. *pulchra*	25,591	40.7	37.2	11.1	10.9	77.9	22.0	0.045	0.011
PCGs									
*A*. *flabellatum*	19,989	33.2	45.9	11.8	9.1	79.0	21.0	−0.161	0.131
*D. binghamiae*	16,470	32.8	44.4	13.0	9.8	77.2	22.8	−0.151	0.138
*D. japonica*	17,448	32.3	44.8	12.8	10.1	77.1	22.9	−0.161	0.117
*H*. *pulchra*	17,442	33.7	44.8	12.4	9.1	78.5	21.5	−0.142	0.151

**Table 4:** Nucleotide composition and skewness of protein-coding genes in the mitogenome of *A. flabellatum*.

PCG	Length (bp)	Nucleotide Composition (%)						AT Skew	GC Skew
		A	T	G	C	A+T	G+C		
*rpl16*	411	45.0	35.5	11.2	8.3	80.5	19.5	0.118	0.150
*cox1*	1611	28.7	42.7	16.5	12.1	71.4	28.6	−0.197	0.154
*cox2*	792	34.8	39.0	14.3	11.9	73.9	26.1	−0.056	0.092
*cox3*	810	28.0	45.8	15.2	11.0	73.8	26.2	−0.241	0.160
*atp4*	540	43.5	45.2	6.7	4.6	88.7	11.3	−0.019	0.180
*cob*	1140	31.5	43.1	13.2	12.3	74.6	25.4	−0.155	0.034
*orf408*	1227	48.3	38.5	5.5	7.7	86.8	13.2	0.114	−0.173
*nad4L*	303	33.3	45.5	12.9	8.3	78.9	21.1	−0.155	0.219
*sdh3*	387	28.9	57.1	7.0	7.0	86.0	14.0	−0.327	0.000
*sdh2*	762	38.5	39.2	12.3	10.0	77.7	22.3	−0.010	0.106
*nad6*	612	34.3	48.0	10.5	7.2	82.4	17.6	−0.167	0.185
*nad6*	612	34.3	48.0	10.5	7.2	82.4	17.6	−0.167	0.185
*sdh2*	762	38.5	39.2	12.3	10.0	77.7	22.3	−0.010	0.106
*sdh3*	387	28.9	57.1	7.0	7.0	86.0	14.0	−0.327	0.000
*atp9*	231	27.3	39.0	22.1	11.7	66.2	33.8	−0.176	0.308
*rps11*	279	47.7	38.0	9.3	5.0	85.7	14.3	0.113	0.300
*nad3*	366	30.3	49.7	13.7	6.3	80.1	19.9	−0.242	0.370
*nad1*	987	29.0	44.6	16.1	10.3	73.6	26.4	−0.212	0.218
*nad2*	1500	29.7	51.8	9.9	8.5	81.5	18.5	−0.271	0.076
*nad4*	1479	28.4	48.3	13.5	9.8	76.7	23.3	−0.260	0.157
*nad5*	1989	27.0	50.0	13.7	9.4	77.0	23.0	−0.298	0.188
*atp8*	429	39.9	44.8	7.9	7.5	84.6	15.4	−0.058	0.030
*atp6*	768	32.2	47.5	11.2	9.1	79.7	20.3	−0.193	0.103
*orf144*	435	37.5	50.6	7.4	4.6	88.0	12.0	−0.149	0.231
*tatC*	753	30.7	58.0	6.4	4.9	88.7	11.3	−0.308	0.129
*rps12*	417	37.4	39.1	12.5	11.0	76.5	23.5	−0.022	0.061

**Table 5:** Features of mitochondrial protein-coding genes in four Delesseriaceae species.

Genes	Length (bp)	Amino Acids	Start Codon	Stop Codon	Coding Strand
*rpl16*	411 ^a^/405 ^b,c,d^	136 ^a^/134 ^b,c,d^	ATG ^a,b,c,d^	TAA ^a,c,d^/TAG ^b^	H ^a,d^/L ^b,c^
*cox1*	1611 ^a^/1608 ^b,c^/1599 ^d^	536 ^a^/535 ^b,c^/532 ^d^	ATG ^a,b,c,d^	TAA ^a,b,c,d^	H ^a,b,c,d^
*cox2*	792 ^a^/780 ^b,c^/741 ^d^	263 ^a^/259 ^b,c^/246 ^d^	ATG ^a,b,c,d^	TAA ^a,b,c,d^	H ^a,b,c,d^
*cox3*	810 ^a^/630 ^b^/819 ^c,d^	269 ^a^/209 ^b^/272 ^c,d^	ATG ^a,b,c,d^	TAA ^a,b,c,d^	H ^a,b,c,d^
*atp4*	540 ^a,b,c^/528 ^d^	179 ^a,b,c^/175 ^d^	ATG ^a,b,c,d^	TAA ^a,b,c,d^	H ^a,b,c,d^
*cob*	1140 ^a,b,d^/1146 ^c^	379 ^a,b,d^/381 ^c^	ATG ^a,b,c,d^	TAA ^a,c,d^/TAG ^b^	H ^a,b,c,d^
*orf408*	1227 ^a^	408 ^a^	ATG ^a^	TAA ^a^	L ^a^
*nad4L*	303 ^a,b,c,d^	100 ^a,b,c,d^	ATG ^a,b,c,d^	TAA^a,b,c,d^	L^a^/H ^b,c,d^
*sdh3*	387 ^a,d^/390 ^c^	128 ^a,d^/129 ^c^	ATG ^a,c,d^	TAA^a,c,d^	H^a,c^/L ^d^
*sdh2*	762 ^a^/858 ^b^/771 ^c,d^	253 ^a^/285 ^b^/256 ^c,d^	ATG ^a,b,c,d^	TAA ^a^/TAG ^b,c,d^	H ^a,b,c^/L ^d^
*nad6*	612 ^a^/651 ^b^/609 ^c,d^	203 ^a^/216 ^b^/202 ^c,d^	ATG ^a,b,c,d^	TAA ^a,b,c,d^	H ^a^/L ^b,c,d^
*nad6*	612 ^a^	203 ^a^	ATG ^a^	TAA ^a^	L ^a^
*sdh2*	762 ^a^	253 ^a^	ATG ^a^	TAA ^a^	L ^a^
*sdh3*	387 ^a^	128 ^a^	ATG ^a^	TAA ^a^	L ^a^
*atp9*	231 ^a,b,c,d^	76 ^a,b,c,d^	ATG ^a,b,c,d^	TAA ^a,b,c,d^	L ^a,b,c,d^
*rps11*	279 ^a^/516 ^b^/363 ^c,d^	92 ^a^/171 ^b^/120 ^c,d^	GTG ^a^/ATG ^b,c,d^	TAA ^a,b,c,d^	L ^a,b,c,d^
*nad3*	366 ^a,b,c,d^	121 ^a,b,c,d^	ATG ^a,b,c,d^	TAA ^a,b,c,d^	L ^a,b,c,d^
*nad1*	987 ^a^/981 ^b,c,d^	328 ^a^/326 ^b,c,d^	ATG ^a,b,c,d^	TAA ^a,c,d^/TAG ^b^	L ^a,b,c,d^
*nad2*	1500 ^a^/1503 ^b,c^/1506 ^d^	499 ^a^/498 ^b,c^/499 ^d^	ATG ^a,b,c,d^	TAA ^a,b,d^/TAG ^c^	L ^a,b,c,d^
*nad4*	1479 ^a^/1485 ^b,c,d^	492 ^a^/494 ^b,c,d^	ATG ^a,b,c,d^	TAA ^a,b,c,d^	L ^a,b,c,d^
*nad5*	1989 ^a^/1971 ^b,c^/1986 ^d^	662 ^a^/656 ^b,c^/661 ^d^	ATG ^a,b,c,d^	TAA ^a,b,c,d^	L ^a,b,c,d^
*atp8*	429 ^a^/420 ^b,c^/414 ^d^	142 ^a^/139 ^b,c^/137 ^d^	ATG ^a,b,c,d^	TAG ^a^/TAA ^b,c,d^	L ^a,b,c,d^
*atp6*	768 ^a,d^/774 ^b,c^	255 ^a,d^/257 ^b,c^	ATG ^a,b,c,d^	TAA ^a,b,c,d^	L ^a,b,c,d^
*orf144*	435 ^a^	144 ^a^	ATG ^a^	TAA ^a^	L ^a^
*tatC*	753 ^a^/492 ^b^/723 ^c^/789 ^d^	250 ^a^/163 ^b^/240 ^c^/262 ^d^	GTG ^a^/ATG ^b,d^/TTA ^c^	TAA ^a,b,c,d^	H ^a,d^/L ^b,c^
*rps12*	417 ^a^/324 ^b^/321 ^c^/318 ^d^	138 ^a^/107 ^b^/106 ^c^/105 ^d^	ATG ^a,b,c,d^	TAA ^a,b,c,d^	H ^a,d^/L ^b,c^
*sdh4*	246 ^b,c,d^	81 ^b,c,d^	ATG ^b,c,d^	TAA ^b,c,d^	L ^b,c,d^
*rps3*	246 ^b^/693 ^c^/687 ^d^	81 ^b^ /230 ^c^/228 ^d^	ATG ^b,c,d^	TAA ^b,c,d^	L ^b,c^/H ^d^

^a^ *A. flabellatum* (PX111276); ^b^ *D. binghamiae* (KX247283); ^c^ *D. japonica* (OR885330); ^d^ *H. pulchra* (OR885332).

## Data Availability

The genome sequence data that support the findings of this study are openly available in GenBank of NCBI at (https://www.ncbi.nlm.nih.gov/) under accession number PX111276. The associated BioProject, Bio-Sample and SRA numbers were PRJNA1372931, SAMN53630808, and SRR36283300, respectively.
